# AdipoRon, an Adiponectin Receptor Agonist, Promotes Intestinal Epithelial Differentiation in Caco-2 Colorectal Adenocarcinoma Cells

**DOI:** 10.3390/biomedicines14081698

**Published:** 2026-07-28

**Authors:** Marta Mallardo, Furqan Memon, Ludovica D’Auria, Raffaella Pagliaro, Aurora Daniele, Ersilia Nigro

**Affiliations:** 1 CEINGE-Biotecnologie Avanzate Scarl “Franco Salvatore”, Via G. Salvatore 486, 80145 Napoli, Italy; marta.mallardo@unipegaso.it (M.M.); dr.furqan.memon@gmail.com (F.M.); daurial@ceinge.unina.it (L.D.); ersilia.nigro@unicampania.it (E.N.); 2 Dipartimento di Scienze dell’Educazione e dello Sport, Università Telematica Pegaso, Centro Direzionale Isola F2, 80143 Napoli, Italy; 3 Dipartimento di Scienze e Tecnologie Ambientali, Biologiche, Farmaceutiche, Università della Campania “Luigi Vanvitelli”, Via Vivaldi 43, 81100 Caserta, Italy; 4 Dipartimento di Scienze Mediche Traslazionali, Università della Campania “L. Vanvitelli”, 80131 Napoli, Italy; raffy.pagliaro@gmail.com; 5 U.O.C. Clinica Pneumologica L. Vanvitelli, Monaldi Hospital, A.O. dei Colli, 80131 Napoli, Italy; 6 Dipartimento di Medicina Molecolare e Biotecnologie Mediche, Università degli Studi “Federico II”, Via Pansini, 5, 80131 Napoli, Italy

**Keywords:** adiponectin receptor synthetic agonist AdipoRon, intestinal epithelial Caco-2 cell line, proliferation, differentiation, ROS levels

## Abstract

**Background:** Colorectal cancer (CRC) remains one of the most prevalent and lethal malignancies worldwide. Adiponectin, a hormone secreted by adipose tissue, has been increasingly recognized for its pleiotropic effects in several malignancies. In particular, an inverse association between circulating adiponectin levels and CRC incidence supports a potential protective role for adiponectin and its receptors (AdipoR1, AdipoR2). The aim of this study was to investigate the effects of adiponectin on Caco-2 cells, a human CRC cell line, by examining intestinal epithelial differentiation using AdipoRon (AR), a synthetic adiponectin receptor agonist. **Methods:** The effects of AR on Caco-2 cells were assessed by evaluating dome formation and the expression of key molecular markers involved in intestinal epithelial differentiation, including KLF-4, DPPIV, SI, and KRT20, at both transcriptional levels using qRT-PCR and at protein levels using immunofluorescence and Western blot analysis. In addition, mitochondrial reactive oxygen species (ROS) production was assessed using MitoSOX™ Red. **Results:** Our findings showed that AR administration was associated with a dose-dependent increase in AdipoR1 and AdipoR2 expression as well as with dome formation in Caco-2 cells. Furthermore, AR administration reduced Ki-67 expression with no changes in cell cycle and an increase in the differentiation markers DPPIV and SI, while KRT20 remained unchanged. Finally, AR decreased mitochondrial ROS levels during differentiation. **Conclusions:** Our findings provide new evidence that AR contributes to intestinal epithelial differentiation and may represent a potential therapy for colorectal cancer. AdipoRon may contribute to the regulation of intestinal epithelial differentiation and may contribute to restoring epithelial homeostasis in tumor cells. Further research is needed to clarify the underlying mechanisms and assess the translational relevance of adiponectin-based therapies.

## 1. Introduction

Adiponectin, a hormone primarily secreted by adipose tissue, has been increasingly recognized for its pleiotropic beneficial effects that extend beyond metabolic regulation to include anti-inflammatory, anti-proliferative, pro-apoptotic, and anti-angiogenic actions in several malignancies [1]. Its role as a tumor-suppressive factor has been reported in various cancers, such as breast, prostate, and endometrial carcinomas, where low circulating levels of adiponectin are associated with increased cancer risk and poor prognosis [2,3,4,5,6]. Adiponectin exerts its biological effects through two transmembrane receptors, AdipoR1 and AdipoR2, which are widely expressed in many tissues, including the intestinal epithelium [7,8]. Recently, an adiponectin receptor synthetic agonist, AdipoRon (AR), has been produced, which binds to the adiponectin receptors AdipoR1 and AdipoR2 [9].

Colorectal cancer (CRC) remains one of the most prevalent and lethal malignancies worldwide, underscoring the urgent need for novel therapeutic strategies [10]. In Italy, it is the second most frequently diagnosed cancer in women (following breast cancer) and the third in men (following prostate and lung cancer). When considering the total population (men and women combined), it stands as the second most common malignancy in Italy with a 5-year survival rate after diagnosis of 64.2% [11,12]. An inverse association between circulating adiponectin levels and both the incidence and progression of CRC has been reported, supporting a potential protective role for adiponectin and its receptor agonists in colorectal tumorigenesis [13,14,15]. Consistently, in vitro studies have shown that adiponectin inhibits proliferation and induces apoptosis in CRC cell lines, primarily via activation of AMP-activated protein kinase and modulation of key regulators of the cell cycle [16]. Furthermore, we have previously shown that adiponectin reduces the viability and migratory potential of Caco-2 cells, a well-established model of colorectal adenocarcinoma, through the induction of apoptosis, increased oxidative stress, and modulation of the inflammatory profile [17]. Moreover, adiponectin has been shown to reduce inflammatory signaling and β-catenin activity, both of which are central to CRC pathogenesis [18].

Cellular differentiation represents a crucial mechanism for maintaining intestinal homeostasis, and its impairment is a hallmark of colorectal carcinogenesis [19]. Therapeutic strategies, aimed at restoring differentiation programs in cancer cells, have shown promise in limiting proliferation and promoting re-establishment of tissue architecture [20]. Colorectal adenocarcinoma Caco-2 cell line is derived from a colon carcinoma and shows many properties typical of absorptive enterocytes with brush border layer [21]. In this context, dome formation in epithelial cell lines such as Caco-2 cells serves as a functional marker of enterocyte polarization and maturation [22]. Additionally, molecular markers including Dipeptidyl Peptidase IV (DPPIV), Sucrase-Isomaltase (SI), Keratin 20 (KRT20), Krüppel-like factor 4 (KLF-4), and E-cadherin provide valuable insights into distinct stages of epithelial differentiation [23]. While DPPIV and SI reflect brush border enzymatic activity associated with enterocyte maturation, KLF-4 and E-cadherin are key regulators of epithelial identity and cell–cell adhesion [24,25]. Conversely, KRT20 is a cytoskeletal protein often linked to terminal differentiation [26]. The ROS levels during cellular differentiation go through a transient increase that functions as an important signaling mechanism to trigger differentiation programs.

In recent years, the involvement of adiponectin in the hormonal control of differentiation has been demonstrated in several cellular models, particularly in bone marrow mesenchymal stem cells (BMSCs), vascular smooth muscle cells (VSMCs) and skeletal cells, but no data are available on the effects of adiponectin receptor agonist AdipoRon on the differentiation process of colorectal adenocarcinoma [26,27,28,29,30].

Based on these findings, the present study aims to further characterize the biological role of adiponectin in the intestinal tract, specifically focusing on its influence on epithelial differentiation. Using the synthetic adiponectin receptor agonist AdipoRon, we investigated the maturation of Caco-2 cells by evaluating both morphological and molecular hallmarks. Specifically, intestinal differentiation was assessed through the analysis of dome formation, ROS levels, and the expression of key markers—including KLF-4, DPPIV, SI, and KRT20—at both the transcriptional and protein levels.

## 2. Materials and Methods

### 2.1. Cell Culture

The established colorectal adenocarcinoma Caco-2 cell line was kindly provided by the Bank of Human and Animal Continuous Cell Lines-CEINGE Biotecnologie Avanzate “Franco Salvatore”, Napoli, Italy. Cells were grown in Dulbecco’s modified Eagle’s medium (DMEM) (Invitrogen, Carlsbad, MA, USA) supplemented with 20% heat-inactivated fetal bovine serum (FBS) *v*/*v*, 2 mM l-glutamine, penicillin (100 U/mL), and streptomycin (100 μg/mL) and kept in an incubator with 5% CO_2_ at 37 °C. Medium was replaced every 3 days. On day 7 post-confluence, cells were treated, as this time point is appropriate for assessing Caco-2 differentiation [21,31].

Caco-2 cells were stained with trypan blue and seeded at a density of 6 × 10^4^ cells/cm^2^ onto 12-well plates (Corning Life Sciences, Tewksbury, MA, USA) pre-coated with rat tail Type I collagen (BD Biosciences, San Jose, CA, USA). The differentiation process of Caco-2 cells was evaluated 10 days after seeding. On day 7 post-confluence, the cells were treated with AR (5, 10 μg/mL) for 48 h, with the drug being refreshed every 24 h. AR doses were selected on the basis of previously published data [17]. AdipoRon stock solution was dissolved in Dimethyl sulfoxide (DMSO) and subsequently diluted in culture medium to obtain the desired final concentrations. Negative control (NC) cells were treated with the same final concentration of DMSO as the highest AR dose, corresponding to 0.2% (*v*/*v*).

Preliminary time-course treatments (24 h, 48 h, and 72 h) with AR showed that the major effects occurred after 48 h. Therefore, this time point was selected for subsequent analyses.

### 2.2. RNA Extraction and Quantitative Real-Time PCR

Caco-2 cells were seeded at a density of 6 × 10^4^ cells/cm^2^ and total RNA was extracted from Caco-2 cells by using TRIzol Reagent (Thermo Fisher Scientific, Waltham, MA, USA) according to the manufacturer’s instructions. The RNA concentration was quantified by using fluorescence-based detection with Qubit 4 Fluorometer (Thermo Fisher Scientific, Waltham, MA, USA). One microgram of total RNA was subjected to reverse transcription with SuperScript IV VILO Master Mix (Invitrogen, Carlsbad, MA, USA) according to the manufacturer’s instructions. Gene expression analysis was carried out using the C1000 Touch Thermal Cycler (Bio-Rad, Hercules, CA, USA) and PowerUp SYBR Green Master Mix (Applied Biosystems, Waltham, MA, USA). The thermal cycling protocol was previously described [31]. Glyceraldehyde 3-phosphate dehydrogenase (GAPDH) was used as the housekeeping gene, and fold changes were calculated using the 2^−ΔΔCt^ method, as previously reported [32]. Primer sequences used for qRT-PCR are reported in Table 1. Experiments were performed with three independent biological replicates (*n* = 3).

### 2.3. Western Blotting

Caco-2 cells were seeded at a density of 6 × 10^4^ cells/cm^2^ and total protein was extracted from cells using pre-cooled radioimmunoprecipitation assay (RIPA) buffer (Sigma-Aldrich, St. Louis, MO, USA) supplemented with a protease inhibitor cocktail (Abcam, Cambridge, UK). Protein concentrations were determined using the Bradford assay (Bio-Rad, Hercules, CA, USA), and samples were prepared as previously described [33].

An amount of 30 μg of total cellular protein was loaded onto a polyacrylamide gel and separated by SDS-PAGE. Subsequently, proteins were transferred to PVDF membranes (Pierce Biotechnology, Waltham, MA, USA), incubated overnight at 4 °C with the following primary antibodies, diluted 1:1000 according to the manufacturer’s instructions: KLF-4, E-cadherin and GAPDH (Cell Signaling Technology, Danvers, MA, USA), AdipoR1 (Santa Cruz Biotechnology, Dallas, TX, USA; Cat. No. sc-518030) (Mouse monoclonal antibody raised against amino acids 1-70 of human AdipoR1), and AdipoR2 (Phoenix Pharmaceuticals Inc., Burlingame, CA, USA; Cat. No. H-001-2) (Rabbit purified IgG antibody raised against a specific synthetic peptide of human AdipoR2). The next day, the membranes were incubated with secondary antibodies anti-rabbit (1:3000) or anti-goat (1:5000) (Cell Signaling Technology, Danvers, MA, USA). Finally, protein bands were detected using the ChemiDoc XRS system (Bio-Rad, Hercules, CA, USA) with ECL detection reagents (Pierce Biotechnology, Waltham, MA, USA). Experiments were performed in two independent biological replicates.

### 2.4. Evaluation of Dome Formation

Caco-2 cells were seeded at a density of 6 × 10^4^ cells/cm^2^ in 12-well plates and cultured as described above. On the designated day, the cells were treated with increasing concentrations of AR (5, 10 μg/mL) for 48 h, with the drug refreshed every 24 h. Cells incubated with vehicle (0.2% *v*/*v* DMSO) served as the negative control (NC). Dome formation was monitored using the Cell Discoverer 7 system through time-course image acquisitions over a 48 h period (every 12 h), in order to evaluate the dynamic development of domes over time. The number of domes was manually counted under a Zeiss Axioscan. Experiments were performed in three independent biological replicates.

### 2.5. Cell Cycle Analysis

Caco-2 cells were seeded at a density of 6 × 10^4^ cells/cm^2^ in 12-well plates and cultured as described above. On the designated day, the cells were treated with increasing concentrations of AR (5, 10 μg/mL) for 48 h, with the drug refreshed every 24 h. NC cells were treated with the same final concentration of DMSO as the highest AR dose, corresponding to 0.2% (*v*/*v*). The cells were then collected, resuspended in 0.3 mL of pre-cooled 1× PBS. Subsequently, 0.7 mL of −20 °C ethanol was added dropwise while gently vortexing to obtain a final ethanol concentration of 70% (*v*/*v*) and maintained on ice for a minimum of 30 min. Finally, the cells were then pelleted via centrifugation, resuspended in a 1× PBS solution of 2.5 mg/mL propidium iodide (Sigma-Aldrich, St. Louis, MO, USA), RNAse (1 mg/mL) (Sigma-Aldrich, St. Louis, MO, USA) and 0.1% triton X-100 in phosphate-buffered saline and incubated for 1 h before a flow cytometry analysis (BD LSRII; Becton Dickinson, San Diego, CA, USA).

### 2.6. Immunofluorescence and Image Analysis

Cells were seeded at a density of 6 × 10^4^ cells/cm^2^ on glass coverslips and allowed to adhere overnight under standard culture conditions. Cells were then maintained until day 7 post-confluence before treatment with AR for 48 h, as described above. At the designated days, cells were fixed for 10 min at room temperature (RT) with 4% paraformaldehyde (Electron Microscopy Sciences, Morgantown, PA, USA), washed with Phosphate-Buffered Saline (PBS) (3 washes of 5 min each), permeabilized with 0.1% Triton X-100 (*v*/*v*) in PBS for 15 min at RT, and rinsed with PBS at RT. To prevent non-specific binding of the antibodies, cells were pre-incubated for 30 min with normal goat serum (1:10 *v*/*v*) diluted in PBS at RT. Then the cells were blocked using 0.1% Triton 100X (*v/v*) and 5% FBS (*v*/*v*) in PBS 1X for one hour at room temperature. The cells were then incubated overnight at 4 °C with the following primary antibodies diluted 1:200 in 0.1% Triton 100X and 5% FBS in PBS 1X: anti-Ki-67 antibody (ABcam, Cambridge, UK; Cat. Ab16667), anti-KLF-4 antibody (Cell Signaling Technology, Danvers, MA, USA; Cat. 4038) and anti-E-cadherin antibody (Cell Signaling Technology, Danvers, MA, USA; Cat. 14472). The following day, the cells were incubated at RT in the dark for 2 h with the appropriate secondary antibodies diluted 1:1000: Alexa Fluor ^®^ 488 goat (Thermo Fisher Scientific, St. Louis, MA, USA; Cat. A32723TR) and Alexa Fluor ^®^ 594 goat (Thermo Fisher Scientific, St. Louis, MA, USA; Cat. A-11012). Nuclei were counterstained with DAPI according to the manufacturer’s instructions (Thermo Fisher Scientific, St. Louis, MA, USA). Specifically, nuclei counterstained with DAPI were acquired using the DAPI/blue channel, Alexa Fluor 488-labelled signals were acquired using the green/FITC channel, and Alexa Fluor 594-labelled signals were acquired using the red/TRITC channel. Secondary-antibody-only controls were performed in parallel for all immunofluorescence experiments by omitting the primary antibodies and incubating the cells only with the corresponding secondary antibodies. Control images were acquired using the same microscope settings and exposure times as the corresponding experimental images. Representative DAPI, secondary-antibody channel, and merged images for each secondary antibody used are shown in Supplementary Figure S2. Fluorescent signals were acquired with a Zeiss Cell Discoverer 7 microscope. Negative control samples consisted of cells treated with vehicle (DMSO) only as reported in 2.1. Experiments were performed in two independent biological replicates.

### 2.7. Detection of Mitochondrial ROS Using MitoSOX™ Red and Glutathione Levels

Caco-2 cells were seeded at a density of 6 × 10^4^ cells/cm^2^ and maintained until day 7 post-confluence. Cells were then treated with AR at concentrations of 5 and 10 μg/mL for 48 h and mitochondrial ROS were assessed using MitoSOX™ Red according to the manufacturer’s instructions. Briefly, MitoSOX™ Red was prepared as a 5 mM stock solution in DMSO and diluted in phenol-red-free Hank’s Balanced Salt Solution (HBSS) (Thermo Fisher Scientific, St. Louis, MA, USA, cat n. M36008) to a final concentration of 5 μM. Cells were washed with HBSS and incubated with the MitoSOX™ Red working solution for 10 min at 37 °C in the dark. After incubation, cells were washed twice with HBSS to remove excess probe. Fluorescence was visualized using the Cell Discoverer 7 system equipped with appropriate filters (excitation ~510 nm, emission ~580 nm). The red fluorescence signal reflects mitochondrial accumulation of oxidized MitoSOX™ Red, indicating superoxide production. Vehicle-treated cells were used as controls to assess baseline ROS levels.

Glutathione levels were quantified using a Glutathione Assay Kit (Cayman Chemical, Ann Arbor, MI, USA), according to the manufacturer’s instructions. Caco-2 cells were maintained until day 7 post-confluence and treated with AR at 5 and 10 μg/mL for 48 h, with vehicle-treated cells used as negative controls. At the end of treatment, cells were washed with cold PBS, collected, and processed for glutathione measurement. Absorbance was measured at 405–414 nm using a microplate reader, and glutathione concentrations were calculated from a standard curve. Data are expressed as mean ± SD of 3 independent experiments.

### 2.8. Statistical Analysis

Data are presented as the mean ± standard deviation (SD) of independent biological replicates. Statistical analyses were performed using GraphPad Prism 6 software (GraphPad Software, San Diego, CA, USA). Differences between two groups were analyzed using an unpaired two-tailed Student’s *t*-test with Welch’s correction, while comparisons among multiple groups were conducted using one-way ANOVA followed by Tukey’s multiple comparisons test. A *p*-value < 0.05 was considered statistically significant.

## 3. Results

### 3.1. AdipoRon Administration Was Associated with a Dose-Dependent Increase in AdipoR1 and AdipoR2 Expression as Well as with Dome Formation in Caco-2 Cells

**Figure 1:**
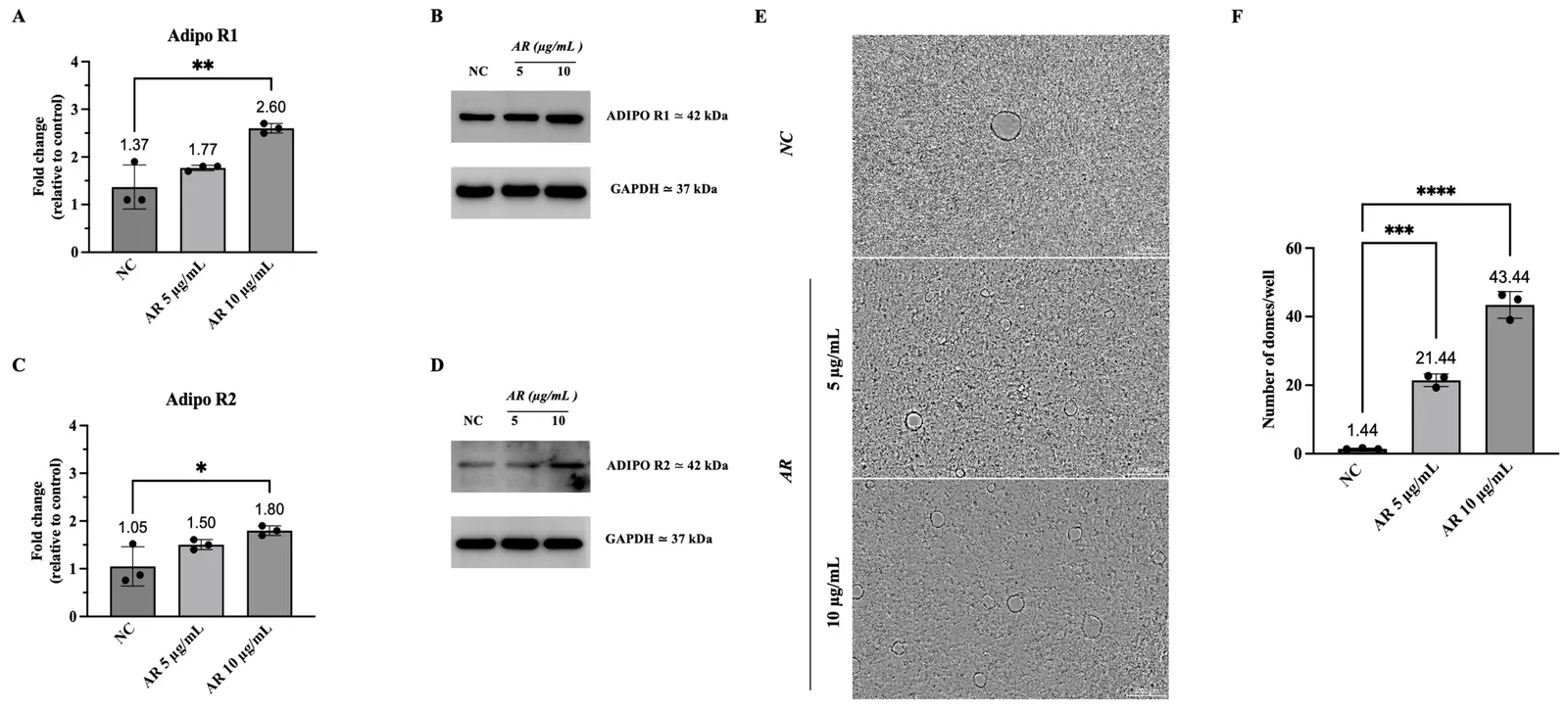
Adiponectin receptor expression is upregulated following 48 h of AdipoRon administration. (**A**,**C**) mRNA expression levels of AdipoR1 and AdipoR2 were assessed after 48 h of AdipoRon treatment in Caco-2 cells. Data were normalized to GAPDH and analyzed using the 2^−ΔΔCt^ method. (**B**,**D**) Western blot analysis was performed using Glyceraldehyde-3-phosphate dehydrogenase (GAPDH) as the housekeeping control and representative blots from two independent experiments are shown. (**E**,**F**) Caco-2 cells were treated with AdipoRon (5 and 10 μg/mL) for 48 h and assessed for dome formation; representative images of domes (**E**) and their quantification (**F**) are shown. The number of domes per well was counted and presented as the mean ± standard deviation (SD) of three independent experiments. NC: negative control (cells treated with vehicle only); AR: AdipoRon. Statistical analysis was performed using one-way ANOVA followed by Tukey’s multiple comparisons test. * *p* < 0.05; ** *p* < 0.01; *** *p* < 0.001; **** *p* < 0.0001. Scale bar = 100 μm.

We confirmed the presence of AdipoRs in Caco-2 cells at both mRNA and protein levels (see Figure 1, panels A–D). On day 7 post-confluence, cells were treated with AR (5 and 10 μg/mL) for 48 h. A significant increase in AdipoR1 and AdipoR2 expression was observed at mRNA levels (Figure 1, panels A and C). The same trend was also observed at protein levels (Figure 1, panels B and D) (*p* < 0.05).

We then investigated whether 48 h of AdipoRon treatment induced functional changes in Caco-2 cells by assessing dome formation. As shown in Figure 1, panels E and F, AR treatment resulted in a dose-dependent increase in dome formation (number), with a 20- and 40-fold increase observed in cells treated with 5 μg/mL or 10 μg/mL AR, respectively (*p* < 0.01). This increase in dome formation, a well-established functional indicator of enterocyte polarization and maturation, suggests that AR promotes intestinal epithelial differentiation.

### 3.2. AdipoRon Administration Reduces the Expression of the Proliferation Marker Ki-67 in Caco-2 Cells

**Figure 2:**
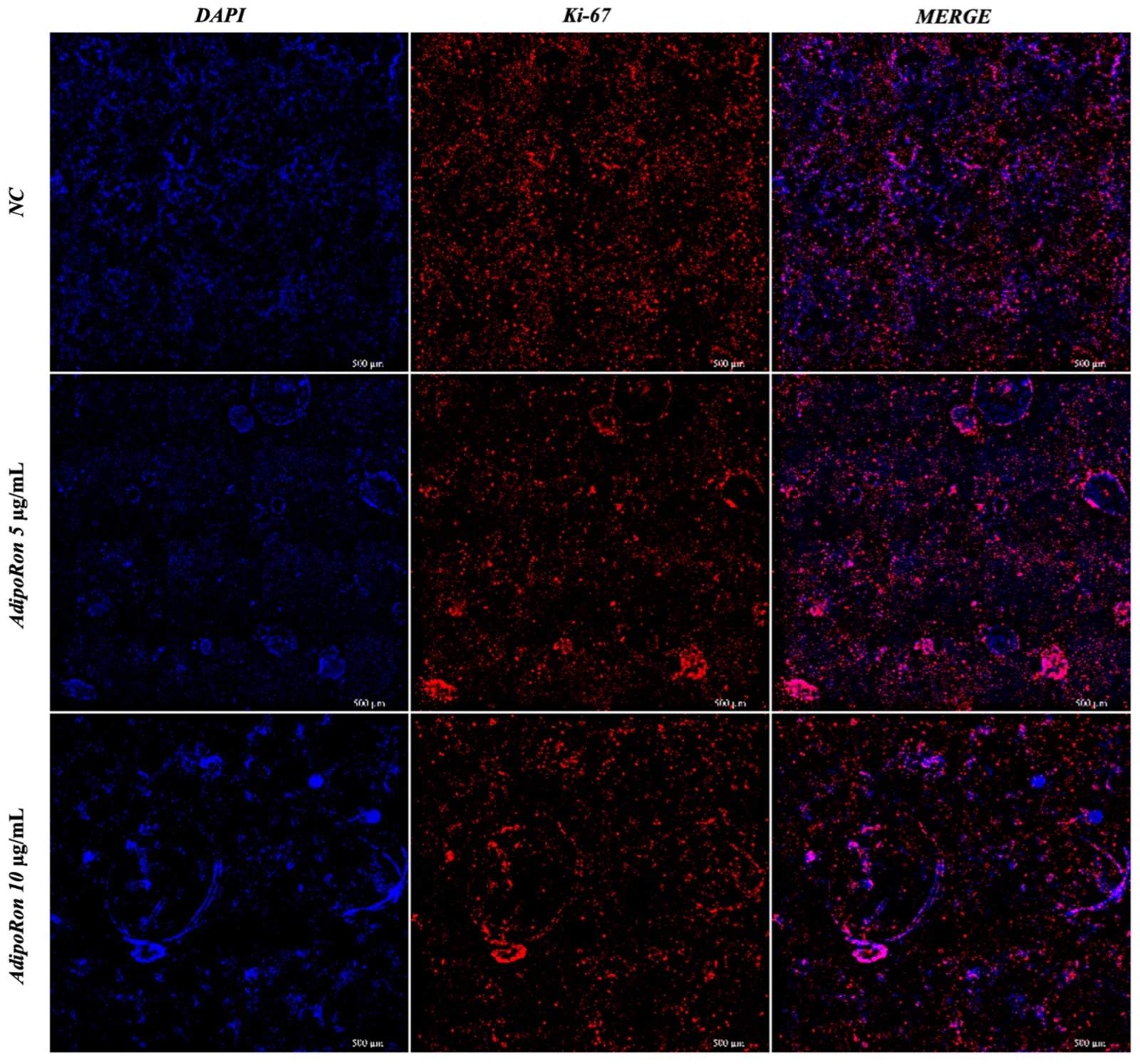
AdipoRon administration downregulates Ki-67 expression in Caco-2 cells. Immunofluorescence analysis of Ki-67 expression in Caco-2 cells treated with AdipoRon for 48 h. Cells were incubated with anti-Ki-67 antibody and then counterstained with DAPI to label the nuclei. Representative images from two independent biological replicates are shown. NC: negative control (cells treated with vehicle only). Scale bar = 500 μm.

Caco-2 cells were cultured and maintained until day 7 post-confluence, then treated with AR (5 and 10 μg/mL) for 48 h. To investigate whether AR may play a role in cell proliferation, we performed immunofluorescence staining for Ki-67, a nuclear protein tightly associated with active cell proliferation. As shown in Figure 2, Ki-67 levels were not affected by treatment with 5 μg/mL AR, whereas a reduction was observed in Caco-2 cells treated with 10 μg/mL AR compared with vehicle controls in two independent biological replicates. Interestingly, the reduction in Ki-67 levels was particularly evident along the margins of the domes. A higher magnification of the staining is shown in Supplementary Figure S3 to visualize the nuclear localization of Ki-67. However, cell-cycle analysis did not reveal substantial changes in the distribution of cells across the cell-cycle phases. Therefore, the reduction in Ki-67 expression might be attributable to a decrease in proliferative activity or apoptosis or cell death rather than to a specific cell-cycle arrest (Supplementary Table S1).

### 3.3. After Administration of AdipoRon, the Expression of the Differentiation Markers DPPIV and SI Increased, While That of KRT20 Does Not Change

**Figure 3:**
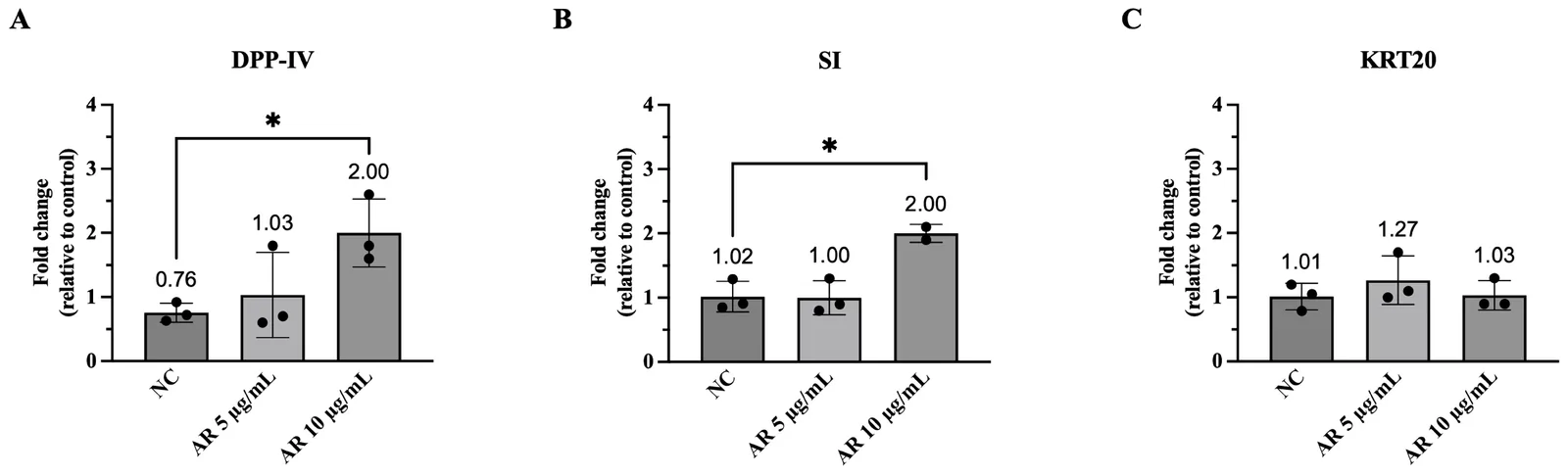
AdipoRon administration upregulates DPPIV and SI differentiation markers in Caco-2 cells, with no effect on Keratin20 (KRT20) expression. (**A**–**C**) mRNA expression levels of (Dipeptidyl Peptidase IV (DPPIV) (**A**), SI (**B**) and KRT20 (**C**) were assessed in Caco-2 cells after 48 h of AdipoRon treatment via qPCR. Data were normalized to Glyceraldehyde-3-phosphate dehydrogenase (GAPDH) and analyzed using the 2^−ΔΔCt^ method. Values are expressed as the mean of three independent experiments ± SD. NC: negative control (cells treated with vehicle only); AR: AdipoRon. Statistical analysis was performed using one-way ANOVA followed by Tukey’s multiple comparisons test. * *p*-value < 0.05.

To further understand the involvement of AR in the differentiation process of Caco-2 cells, we assessed the mRNA expression levels of the differentiation markers DPPIV, SI, and KRT20. Compared with vehicle controls, cells treated with AR at 10 μg/mL showed a 2-fold increase in the mRNA expression levels for DPPIV and SI (Figure 3, panels A and B) (*p* < 0.05 and *p* < 0.01, respectively). In contrast, the mRNA levels for KRT20 remained unchanged after AR treatment (Figure 3, panel C). The increased expression of DPPIV and SI supports the hypothesis that AR promotes the differentiation of Caco-2 cells.

**Figure 4:**
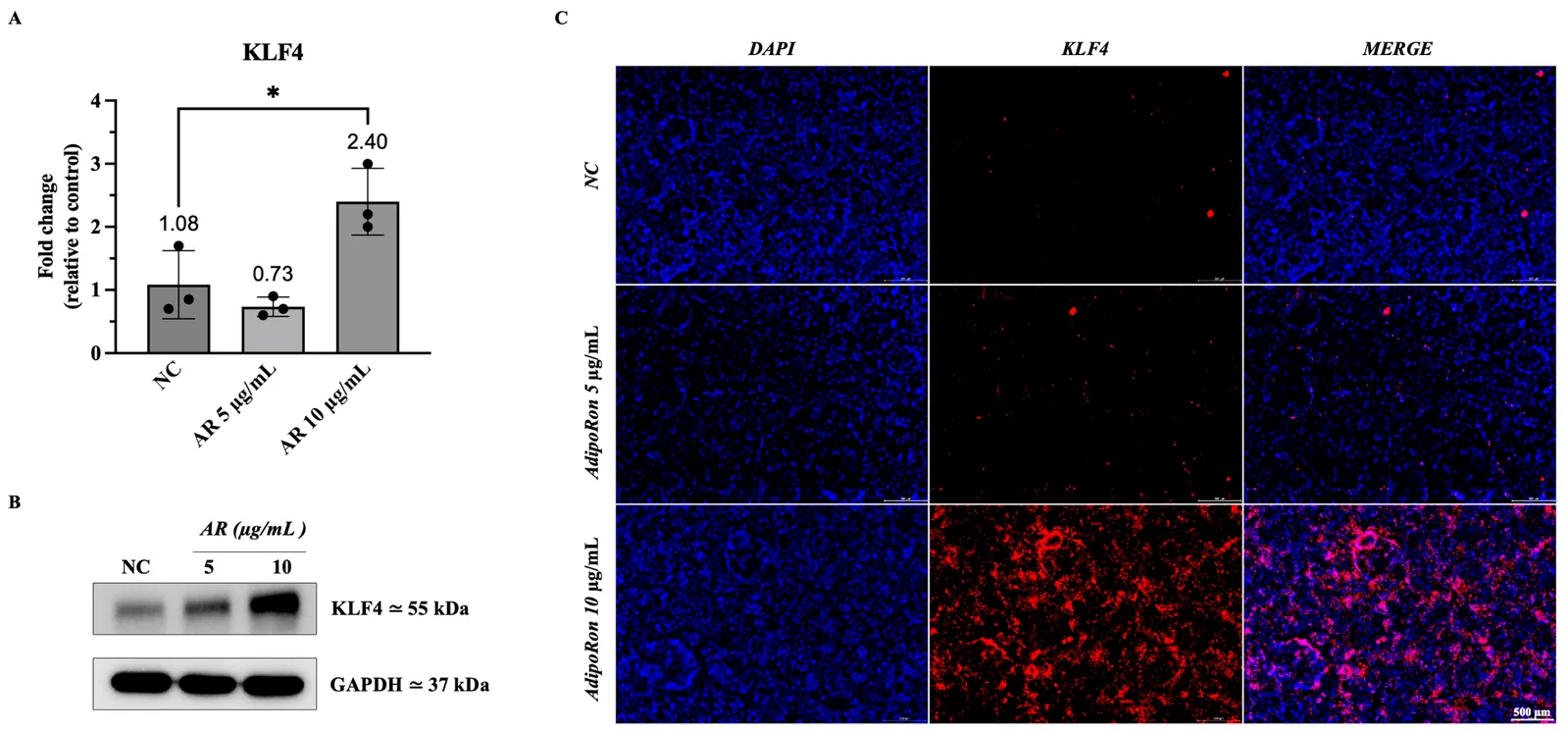
AdipoRon upregulates differentiation marker Krüppel-like factor 4 (KLF-4) in Caco-2 cells. (**A**) mRNA expression levels of KLF-4 were evaluated after 48 h of AdipoRon treatment in Caco-2 cells using qPCR. Data were normalized to Glyceraldehyde-3-phosphate dehydrogenase (GAPDH) and analyzed using the 2^−ΔΔCt^ method and presented as the mean ± standard deviation (SD) of three independent experiments. (**B**) Western blot analysis was performed using GAPDH as the housekeeping control and representative blots from two independent biological replicates are shown. (**C**) Immunofluorescence staining of KLF-4; the cells were incubated with anti-KLF-4 antibody and then counterstained with DAPI to label the nuclei. NC: negative control (cells treated with vehicle only); AR: AdipoRon. Statistical analysis was performed using one-way ANOVA followed by Tukey’s multiple comparisons test. * *p* < 0.05. Scale bar = 500 μm.

Next, given the crucial roles of KLF-4 and E-cadherin in regulating cell differentiation and adhesion, we investigated whether AR treatment could modulate their expression levels. Our results revealed that exposure to 10 μg/mL AR resulted in a significant 2-fold increase in KLF-4 mRNA expression compared to vehicle controls (Figure 4, panel A) (*p* < 0.05). This upregulation was also observed at the protein level by Western blotting and immunofluorescence analysis in two independent biological replicates (Figure 4, panels B and C).

**Figure 5:**
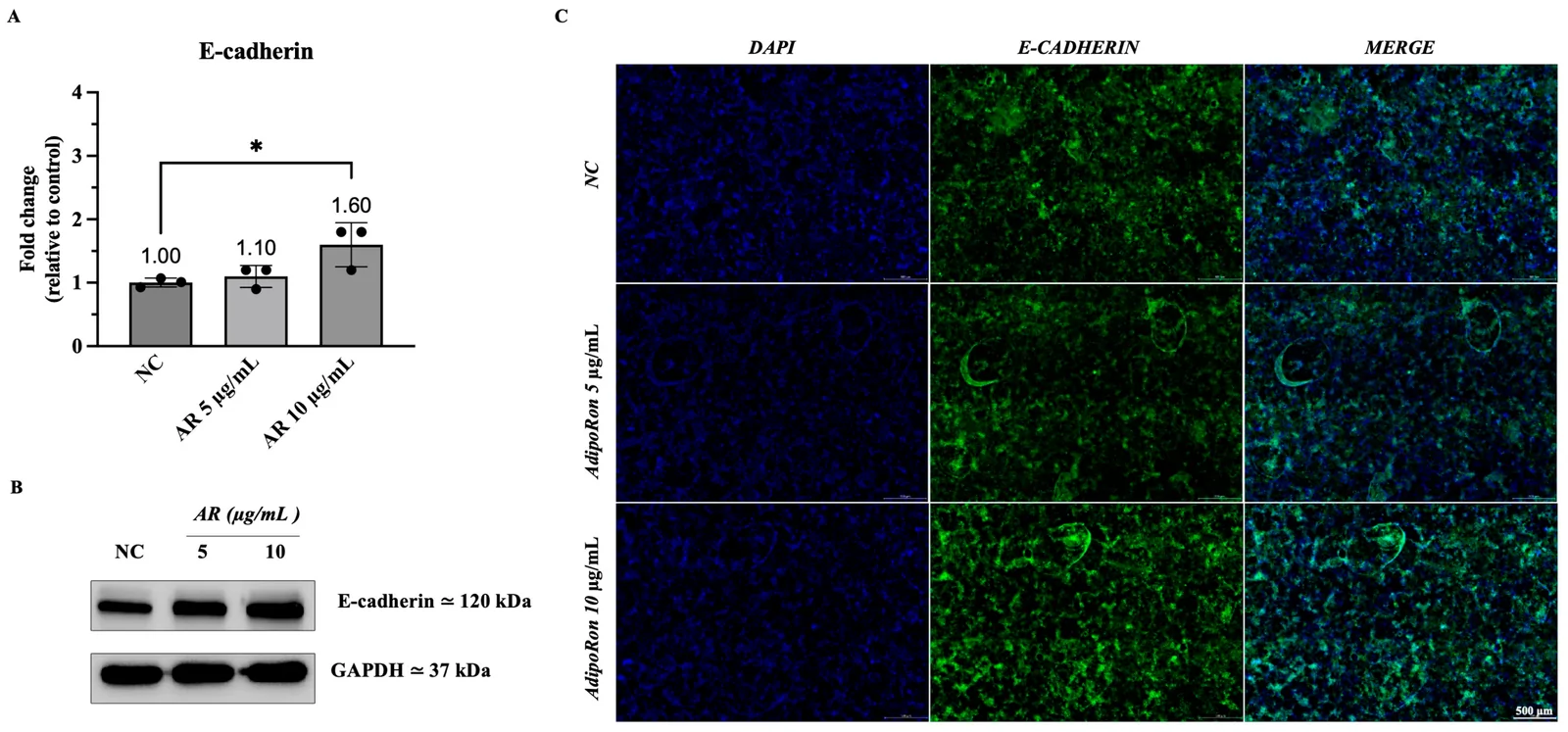
AdipoRon administration upregulates E-cadherin expression in Caco-2 cells. (**A**) mRNA expression levels of E-cadherin were evaluated after 48 h of AdipoRon treatment in Caco-2 cells using qPCR. Data were normalized to Glyceraldehyde-3-phosphate dehydrogenase (GAPDH) and analyzed using the 2^−ΔΔCt^ method and presented as the mean ± standard deviation (SD) of three independent experiments. (**B**) Western blot analysis was performed using GAPDH as the housekeeping control and representative blots from two independent biological replicates are shown. (**C**) Immunofluorescence staining of E-cadherin; the cells were incubated with anti-E-cadherin antibody and then counterstained with DAPI to label the nuclei. NC: negative control (cells treated with vehicle only); AR: AdipoRon. Statistical analysis was performed using one-way ANOVA followed by Tukey’s multiple comparisons test. * *p* < 0.05. Scale bar = 500 μm.

Similarly, E-cadherin mRNA levels were significantly upregulated after treatment with 10 μg/mL AR (Figure 5, panel A) (*p* < 0.05), with a corresponding increase in its protein levels, as shown by Western blotting and immunofluorescence analysis (Figure 5, panels B and C).

**Figure 6:**
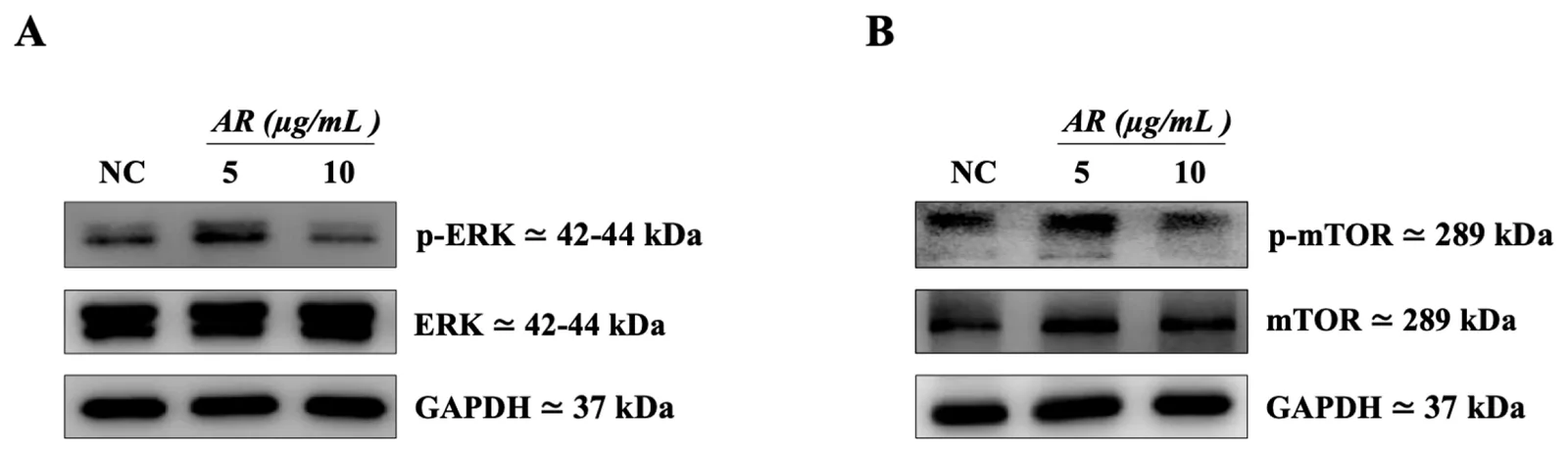
AdipoRon suppresses ERK and mTOR signaling in Caco-2 cells. (**A**) Western blot analysis showing reduced phosphorylation of ERK (p-ERK) and (**B**) mTOR (p-mTOR) in Caco-2 cells treated with AdipoRon (5 and 10 μg/mL) for 48 h compared to control cells. GAPDH was used as a loading control. Representative blots from two independent biological replicates are shown. NC: negative control (cells treated with vehicle only); AR: AdipoRon.

To investigate the molecular mechanisms underlying the differentiation-promoting effects of AR, we evaluated the activation status of ERK and mTOR signaling pathways by Western blot analysis. As shown in Figure 6, 10 μg/mL AR treatment resulted in a reduction in the phosphorylation levels of ERK and mTOR compared to vehicle controls. These findings indicate that AR suppresses ERK and mTOR signaling pathways, which are known to regulate cell proliferation and differentiation, and suggest that modulation of these pathways may contribute to the induction of epithelial differentiation observed in Caco-2 cells.

### 3.4. AdipoRon Induces a Reduction in Mitochondrial ROS Levels Concomitant with Dome Formation

**Figure 7:**
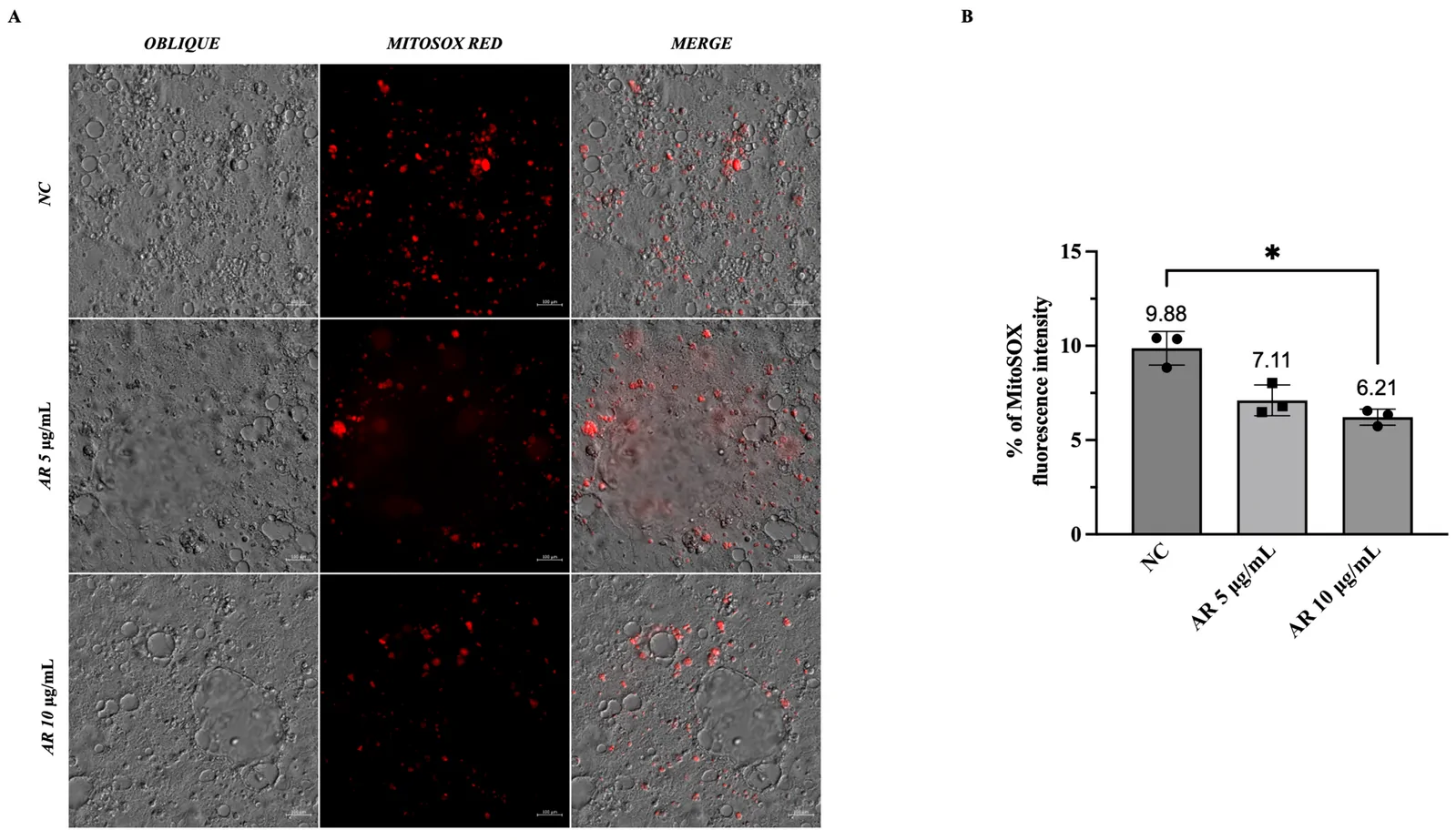
Detection of mitochondrial Reactive Oxygen Species (ROS) using MitoSOX Red in Caco-2 cells. Caco-2 cells were maintained until day 7 post-confluence and then treated with AdipoRon (5, 10 μg/mL) for 48 h and stained with MitoSOX Red. (**A**) The fluorescence intensity was visualized using fluorescence microscopy, highlighting the presence of mitochondrial ROS. The red signal corresponds to the accumulation of MitoSOX Red in the mitochondria, indicating superoxide production. (**B**) quantitation of the signal. Statistical analysis was performed using one-way ANOVA followed by Tukey’s multiple comparisons test. Data are expressed as mean ± SD. * *p* < 0.05. Scale bar = 100 μm.

Since mitochondrial ROS production can influence cell cycle progression and/or differentiation, we assessed the impact of AR on mitochondrial ROS production. As shown in Figure 7, the mean intensity decreased from 9.88 in control cells to 7.11 in cells treated with 5 μg/mL AR and to 6.21 in cells treated with 10 μg/mL AdipoRon. The reduction was statistically significant for AR-treated cells compared with control cells (* *p* < 0.05). These results suggest that AR reduces mitochondrial ROS levels, potentially contributing to a more favorable cellular environment for differentiation. In addition, quantitative analysis of glutathione levels revealed that AR increased the levels compared to control, further confirming an enhancement of the intracellular antioxidant capacity (Supplementary Figure S1).

## 4. Discussion

The present study demonstrates that the synthetic adiponectin receptor agonist AR promotes intestinal epithelial differentiation in Caco-2 colorectal adenocarcinoma cells. AdipoRon treatment induced a dose-dependent increase in dome formation, a well-established hallmark of epithelial polarization, and was accompanied by upregulation of key differentiation markers, including DPPIV, SI, KLF-4, and E-cadherin. Notably, these effects were associated with a reduction in the proliferation marker Ki-67, supporting the notion that AR shifts Caco-2 cells from a proliferative toward a more differentiated epithelial phenotype.

Although adiponectin has been extensively studied in cellular models of colon carcinoma, to our knowledge, no data are currently available on the role of this adipokine in the process of colon cell differentiation in Caco-2 cells. Here, we investigated the effects of AR on Caco-2 cellular proliferation and differentiation, clearly demonstrating its direct effect on cell differentiation by significantly increasing dome formation. This phenomenon was associated with increased expression of AdipoR1 and AdipoR2, which may indicate a self-reinforcing regulatory mechanism, in which activation of adiponectin signaling enhances receptor expression, thus amplifying downstream effects. Consistently, AR has been shown to increase AdipoR1 and AdipoR2 expression in different models, including diabetic kidney, retinal, and B-cell leukemia cells [34,35]. Although these findings derive from non-intestinal models, they support the idea that AR not only activates but may also sensitize cells to adiponectin signaling by upregulating receptor levels. The concomitant reduction in the proliferation marker Ki-67, accompanied by the induction of differentiation markers and dome formation, suggests that AR could promote a shift from a proliferative to a more differentiated phenotype in Caco-2 cells. However, since cell-cycle analysis did not show substantial changes in the distribution of cells across the major cell-cycle phases, and since specific quiescence markers were not evaluated, we cannot conclude that AR induces G0 arrest or cellular quiescence. Accordingly, the Ki-67 data should be interpreted as evidence of reduced proliferative activity associated with differentiation, rather than as direct evidence of cell-cycle arrest. Indeed, proliferating cells are typically less differentiated, whereas non-proliferating cells display a more mature phenotype [36,37]. The literature data report that in monolayer models of colon cancer epithelial cells, adiponectin exerts antiproliferative effects [17]. However, to our knowledge, this is the first evidence showing that an adiponectin receptor agonist is able to induce differentiation in Caco-2 cells, whereas its pro-differentiation activity has been described only in other cellular models such as mesenchymal stem cells (BMSCs), smooth muscle cells (VSMCs), preadipocytes and skeletal myoblasts [27,29,30,38].

We also found that AR treatment upregulated the levels of the differentiation markers DPPIV and SI in Caco-2 cells. Previous studies, although not clarifying the direct regulatory effects of adiponectin on DPPIV expression, suggest a potential relationship between these two proteins, i.e., DPPIV inhibitors such as sitagliptin and vildagliptin have been shown to increase circulating adiponectin levels, probably through modulation of incretin signaling and glucose metabolism [39]. Furthermore, DPPIV itself has been identified as an adipokine [40], capable of impairing insulin sensitivity through autocrine and paracrine mechanisms. Therefore, further investigations are needed to evaluate a possible crosstalk between adiponectin and DPPIV in the regulation of intestinal epithelial maturation and differentiation. Regarding SI, it is a classical marker of mature enterocytes, typically upregulated during the differentiation process [41]. To our knowledge, there is currently no direct evidence that confirms the link between adiponectin and regulation of SI expression. Moreover, we examined KLF-4, as it is a key regulator of intestinal epithelial differentiation and acts as a tumor suppressor by inducing cell cycle arrest and promoting terminal differentiation [42]. The increased expression of KLF-4 observed following AR exposure supports a differentiation-promoting role for adiponectin. To our knowledge, no data are currently available on the direct regulation of KLF-4 by adiponectin, although KLF-4 has been reported to play an important role in adipogenesis [43]. Since epithelial differentiation is closely linked to the maintenance of cell–cell adhesion and polarity, we also investigated E-cadherin, a major component of adherent junctions and a hallmark of epithelial integrity [23] and found that its expression was markedly increased following AR treatment. A relationship between adiponectin and E-cadherin has been largely outlined in the literature. In some cancer models, i.e., breast cancer and lymphoma, adiponectin enhances E-cadherin expression [44]. Similarly, in non-small-cell lung carcinoma (NSCLC), adiponectin has been shown to inhibit migration and invasion by reversing the epithelial–mesenchymal transition (EMT) process; this effect was associated with E-cadherin upregulation and vimentin downregulation [45]. More specifically, in colorectal carcinoma, adipocytokine- and glucocorticoid metabolism-related genes have been shown to be overexpressed in adenocarcinoma tissues, and this overexpression was associated with E-cadherin downregulation, linking these genes to colorectal carcinogenesis and tumor progression [46].

KRT20, a cytoskeletal protein involved in epithelial stress protection and differentiation [47], remained unchanged after AR exposure. This lack of modulation, despite the increase in other differentiation markers, suggests, although not conclusively, that AR may mainly affect early-to-intermediate stages of enterocyte maturation rather than terminal differentiation [42,47,48]. This is consistent with data suggesting that KRT20 expression is more closely associated with terminal cytoskeletal remodeling [42,48]. Therefore, AR may modulate early to intermediate stages of the differentiation program, sufficient to restore epithelial polarity.

When observing the multi-layered structures and the peripheral cells surrounding the fully formed domes, a clear morphological and fluorescence intensity gradient can be appreciated, showing a relative reorganization of some of the above-mentioned markers (i.e., Ki-67, E-cadherin) at the dome edges compared to the dome body. The dome edges are regions characterized by receptor enrichment and continuous cell borders [49], reinforcing the specificity of adiponectin regulation of Caco-2 differentiation.

The ERK and mTOR pathways are key regulators, functionally interconnected, of intestinal epithelial cell fate, controlling the balance between proliferation and differentiation. In particular, inhibition of mTOR signaling promotes epithelial differentiation, polarization, and expression of enterocyte maturation markers in Caco-2 cells [50]. ERK signaling plays a key role in regulating the balance between proliferation and differentiation in intestinal epithelial cells. In Caco-2 cells, ERK activity is elevated during the proliferative phase and decreases as cells undergo spontaneous enterocytic differentiation. The downregulation of ERK and mTOR signaling suggests that such modulation may contribute to the differentiation-promoting effects observed following AR treatment [51].

The levels of ROS during cellular differentiation can either increase or decrease, depending on the cell type and stage of differentiation. In many models, a transient increase in ROS serves as an important signaling mechanism to trigger differentiation programs. To date, no studies have specifically examined ROS dynamics during physiological differentiation of Caco-2 cells (e.g., dome formation and polarization). Here, interestingly, mitochondrial ROS levels decreased in parallel with dome formation after AR treatment, which could reflect a shift toward a more stable redox state that, while promoting differentiation, minimizes proliferative stress [52]. This observation is consistent with the antioxidant role of adiponectin, which has been reported to activate PGC-1α and MnSOD expression [52]. Additionally, quantitative analysis revealed that AdipoRon-treated cells showed increased glutathione levels compared with vehicle controls, further confirming an enhancement of the intracellular antioxidant capacity (Supplementary Figure S1). This concerted robust activation of antioxidant defenses and the concomitant reduction in mitochondrial ROS may therefore represent both a marker and a mediator of epithelial redifferentiation, linking metabolic adaptation to structural maturation. The reduction in mitochondrial ROS may therefore represent both a marker and a mediator of epithelial redifferentiation, linking metabolic adaptation to structural maturation.

This study has several methodological and biological limitations that should be acknowledged. First, our findings were obtained using a single in vitro model (Caco-2 cells), which, although widely used to study intestinal epithelial differentiation, may not fully recapitulate the complexity and heterogeneity of colorectal tumors in vivo. Second, although we observed reduced phosphorylation of ERK and mTOR following AR treatment, we did not employ specific pharmacological inhibitors or genetic approaches to directly demonstrate the causal involvement of these or additional (AMPK) signaling pathways in mediating the differentiation-promoting effects of AR. Further studies using organoids and animal models will be required to determine whether AR exerts similar differentiation-promoting and antiproliferative effects in a complex tissue environment and to assess its potential translational relevance for colorectal cancer therapy. Additionally, gene expression analysis was normalized using a single housekeeping gene; although its stability was verified under our experimental conditions, the use of multiple reference genes should be considered. Finally, we acknowledge that functional proliferation assays, such as EdU incorporation, BrdU assay, or clonogenic assays, were not performed. Future studies will be required to functionally assess the impact of AR on DNA synthesis, cell proliferation rate, and long-term colony-forming ability.

## 5. Conclusions

In conclusion, these findings provide new evidence that AR promotes a hormonal control of intestinal epithelial differentiation. By enhancing differentiation and reducing proliferation, AR seems to act as a molecular reprogramming agent capable of restoring epithelial homeostasis in tumor cells. Disruption of the balance between proliferation and differentiation is a key event in tumorigenesis, especially in rapidly renewing tissues such as the intestinal epithelium. This dual action could therefore represent an attractive strategy to modulate tumor progression. Further investigations in three-dimensional cultures, in organoids and in vivo models are needed to clarify the underlying signaling mechanisms and to assess the translational relevance of adiponectin-based therapies.

## Figures and Tables

**Table 1:** Primer sequences used for qRT-PCR experiments.

*Target Gene*	*Primer Sequence (5′-3′)*
*Dipeptidyl Peptidase IV (DPPIV)*	F: GGCACCTGGGAAGTCATCGGGA
	R: AGAGGGGCAGACCAGGACCG
*Sucrase-Isomaltase (SI)*	F: CATCCTACCATGTCAAGAGCCAG
	R: GCTTGTTAAGGTGGTCTGGTTTAAATT
*Keratin 20 (KRT20)*	F: GGTGAACTATGGGAGCGATCT
	R: CTAGACGGTCATTTAGGTTCTGC
*Krüppel-like factor 4 (KLF-4)*	F: ATCTTTCTCCACGTTCGCGTCTG
	R: AAGCACTGGGGGAAGTCGCTTC
*E-cadherin*	F: AGGCCTCTACGGTTTCATAA
	R: CTTGCCTTCTTTGTCTTTGTT
*Glyceraldehyde-3-phosphate dehydrogenase (GAPDH)*	F: AGCCACATCGCTCAGACAC
	R: GCCCAATACGACCAAATCC

## Data Availability

The original contributions presented in this study are included in the article/Supplementary Materials. Further inquiries can be directed to the corresponding author.

## Supplementary Materials

The following supporting information can be downloaded at: https://www.mdpi.com/article/10.3390/biomedicines14081698/s1.

## Abbreviations

**Abbreviation**	**Meaning**
NC	Negative Control (vehicle-treated cells, 0.2% *v*/*v* DMSO)
DMSO	Dimethyl Sulfoxide
AR	AdipoRon
CRC	Colorectal Cancer
ROS	Reactive Oxygen Species
qRT-PCR	Quantitative Real-Time PCR
SI	Sucrase-Isomaltase
KRT20	Keratin 20
KLF-4	Krüppel-like factor 4
ERK	Extracellular signal-Regulated Kinase
mTOR	Mammalian Target of Rapamycin
Ki-67	Marker of cellular proliferation
