# Supplementary material for: AdipoRon, an Adiponectin Receptor Agonist, Promotes Intestinal Epithelial Differentiation in Caco-2 Colorectal Adenocarcinoma Cells
Source: Biomedicines. 2026 Jul 28;14(8):1698. doi: 10.3390/biomedicines14081698 (PMC13509452; doi:10.3390/biomedicines14081698)
Supplement: Supplementary file 1 [file biomedicines-14-01698-s001.zip › biomedicines-4225739-supplementary.pdf]

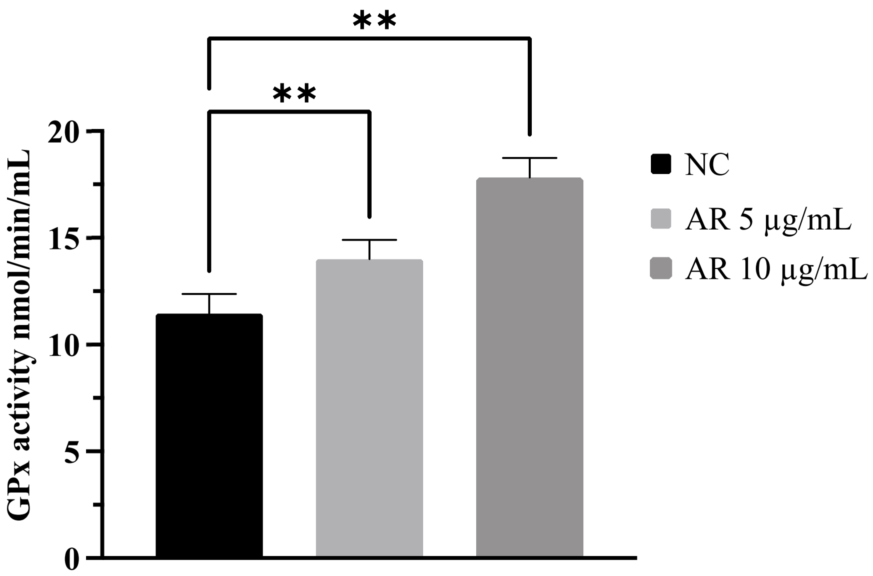

Supplementary Figure S1. Glutathione levels in Caco-2 cells following AdipoRon treatment. Caco-2 cells were maintained until day 7 post-confluence and treated with AdipoRon at 5 and 10 µg/mL for 48 h. Glutathione levels were measured as an indicator of cellular antioxidant/redox status. An increase in glutathione levels was observed in AdipoRon-treated cells compared with vehicletreated control cells. Data are expressed as mean ± SD of independent experiments. NC: negative control (vehicle control), cells treated with vehicle only; AR: AdipoRon. Statistical analysis was performed using one-way ANOVA followed by Tukey's multiple comparisons test. ** $p < 0.01$.

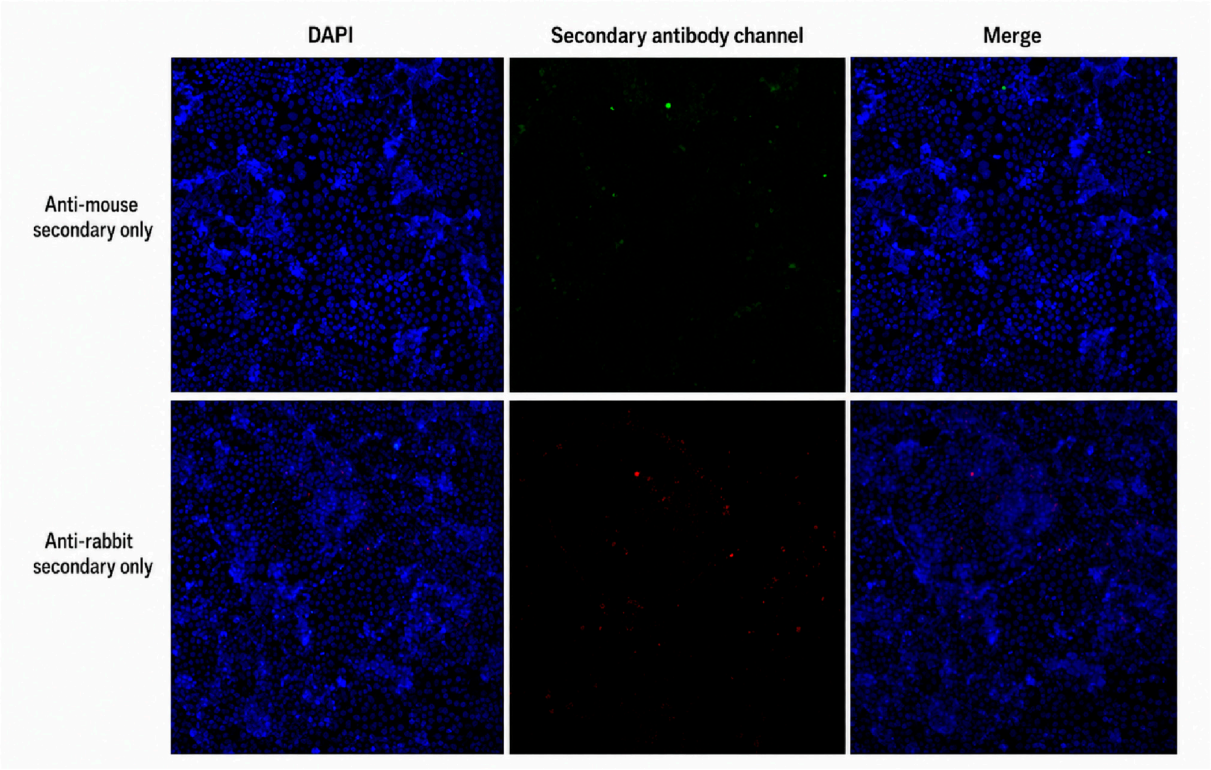

Supplementary Figure S2. Secondary-antibody-only controls for immunofluorescence analysis. Representative secondary-antibody-only negative controls used for the immunofluorescence experiments. Primary antibodies were omitted, and samples were incubated only with the corresponding fluorophore-conjugated secondary antibodies used in the manuscript. Upper panels: DAPI staining, anti-mouse secondary antibody channel, and merged image. Lower panels: DAPI staining, anti-rabbit secondary antibody channel, and merged image.

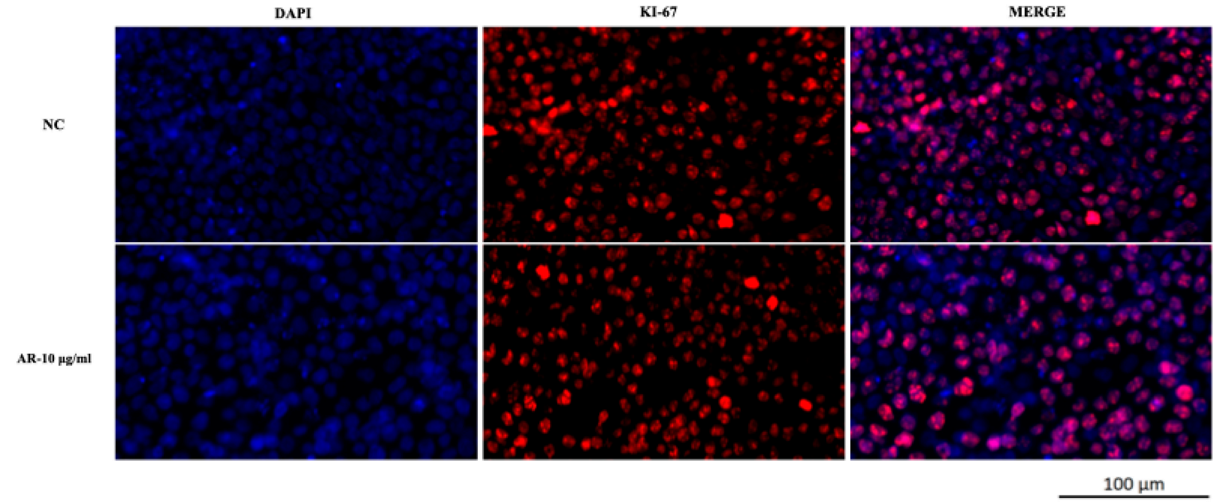

Supplementary Figure S3. Higher magnification of Ki-67 staining. AdipoRon administration downregulates Ki-67 expression in Caco-2 cells. Immunofluorescence analysis of Ki-67 expression in Caco-2 cells treated with AdipoRon for 48 h. Cells were incubated with anti-Ki-67 antibody and then counterstained with DAPI to label the nuclei. Representative images from two independent biological replicates are shown. NC: negative control (cells treated with vehicle only). Scale bar = 100 µm.

Supplementary Table S1.

Group	G1 (%)	S (%)	G2 (%)
**NC**	88,93	10,74	0,32
**AdipoRon 5 µg/mL**	83,83	13,93	2,23
**Adiporon 10 µg/mL**	85,18	13,18	1,63
